# Crystal structure analysis of ethyl 6-(4-meth­oxy­phen­yl)-1-methyl-4-methyl­sulfanyl-3-phenyl-1*H*-pyrazolo­[3,4-*b*]pyridine-5-carboxyl­ate

**DOI:** 10.1107/S2056989020008841

**Published:** 2020-07-07

**Authors:** H. Surya Prakash Rao, Ramalingam Gunasundari, Jayaraman Muthukumaran

**Affiliations:** a Department of Chemistry and Biochemistry, School of Basic Sciences and Research, Sharda University, Greater Noida 201306, India; b Department of Chemistry, Pondicherry University, Puducherry 605 014, India; c Department of Biotechnology, School of Engineering and Technology, Sharda University, Greater Noida 201306, India

**Keywords:** crystal structure, pyrazolo­pyridine, pyrazolo­[3,4-*b*]pyridine, C—H⋯*π* inter­actions

## Abstract

In the title compound, C_24_H_23_N_3_O_3_S, the dihedral angle between the fused pyrazole and pyridine rings is 1.76 (7)°. The benzene and meth­oxy phenyl rings make dihedral angles of 44.8 (5) and 63.86 (5)°, respectively, with the pyrazolo­[3,4-*b*] pyridine moiety. An intra­molecular short S⋯O contact [3.215 (2) Å] is observed. The crystal packing features C—H⋯*π* inter­actions.

## Abstract 2

In the title compound, the dihedral angle between the fused pyrazole and pyridine rings is 1.76 (7)°. The benzene and meth­oxy phenyl rings make dihedral angles of 44.8 (5) and 63.86 (5)°, respectively, with the pyrazolo­[3,4-*b*] pyridine moiety. An intra­molecular short S⋯O contact [3.215 (2) Å] is observed. The crystal packing features C—H⋯*π* inter­actions.

## Chemical context   

Pyrazolo­pyridines, in which a group of three nitro­gen atoms is incorporated into a bicyclic heterocycle, are privileged medicinal scaffolds, often utilized in drug design and discovery regimes (Kumar *et al.*, 2019 ▸). Owing to the possibilities of the easy synthesis of a literally unlimited number of a combinatorial library of small organic mol­ecules with a pyrazolo­pyridine scaffold, there has been enormous inter­est in these mol­ecules among medicinal chemists (Kumar *et al.* 2019 ▸; Pinheiro *et al.* 2019 ▸; Hardy, 1984 ▸). Indeed, mol­ecules with pyrazolo­pyridine in the core structure exhibit multifaceted medicinal properties such as anti-microbial, anti-viral, anti-fungal, anti-hypertensive, analgesic, anti­quorum-sensing, anti-cancer, anti-inflammatory, anti-Alzheimer’s, anti-diabetic, anti-nociceptive, anti-tuberculosis and anti-leishmanial activities (Hardy, 1984 ▸; Hawas *et al.*, 2019 ▸; de Mello *et al.*, 2004 ▸; El-Gohary *et al.*, 2019 ▸; El-Gohary & Shaaban, 2018 ▸). Moreover, pyrazolo­pyridine-derived drug mol­ecules exhibit anti-cancer properties (Huang *et al.*, 2007 ▸; Ye *et al.*, 2009 ▸). They are inhibitors of several important proteins, namely cycline-dependent kinase1, HIV reverse transcriptase, leucine zipper kinase, protein kinase, xanthine oxidase, inter­leukin-6 (IL-6), tumor necrosis factor alpha (TNF-α), phospho­diesterase-4, NAD(P)H oxidases (Kumar *et al.*, 2019 ▸; Gökhan-Kelekçi *et al.*, 2007 ▸; Fathy *et al.*, 2015 ▸; Park *et al.*, 2017 ▸). A recent study reported that they could be promising inhibitors against the enzyme pantothenate synthetase from *Mycobacterium tuberculosis* (Amaroju *et al.*, 2017 ▸). FDA-approved drugs incorporating the pyrazolo­pyridine scaffold include cartazolate, tracazolate and etazolate (Hawas *et al.*, 2019 ▸). Among pyrazolo­pyridines, pyrazolo­[3,4-*b*]pyridines are medicinally important because of the ease of combinatorial library synthesis, adherence to the Lipinski rule and favourable ADMET properties. (Chauhan & Kumar, 2013 ▸; Zhai *et al.*, 2019 ▸). Based on the importance of pyrazolo­[3,4-*b*]pyridine-containing mol­ecules, we have undertaken a single-crystal X-ray diffraction study of the title compound. We have recently analyzed the solid-state structure of a pyrazolo­[3,4-*b*]pyridine-containing mol­ecule, ethyl 3-(4-chloro­phen­yl)-1,6-di­methyl-4-meth­ylsulfanyl-1*H*-pyrazolo­[3,4-b]pyridine-5-carb­ox­ylate (NUDWOB; Rao *et al.* 2020 ▸), but the title compound exhibits a very different conformational structure of the substituents and supra­molecular structure, as discussed here.
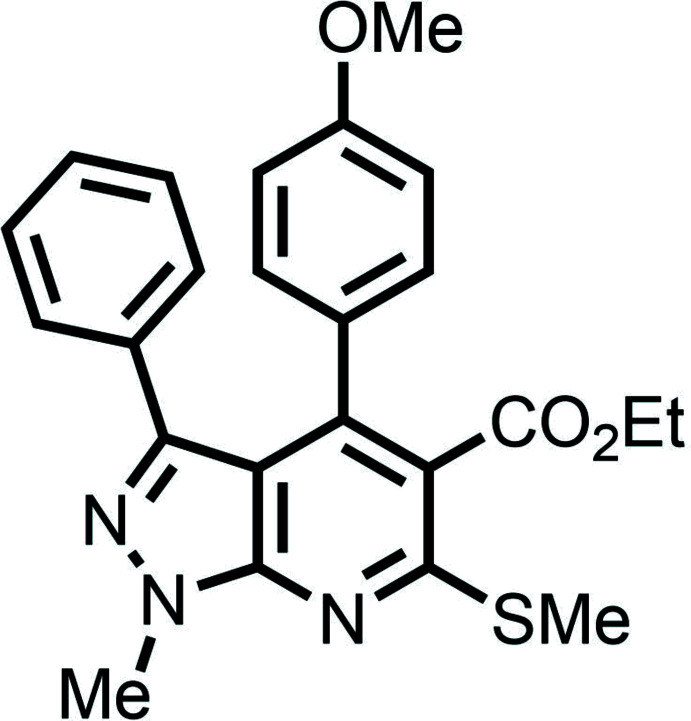


## Structural commentary   

**Figure 1:**
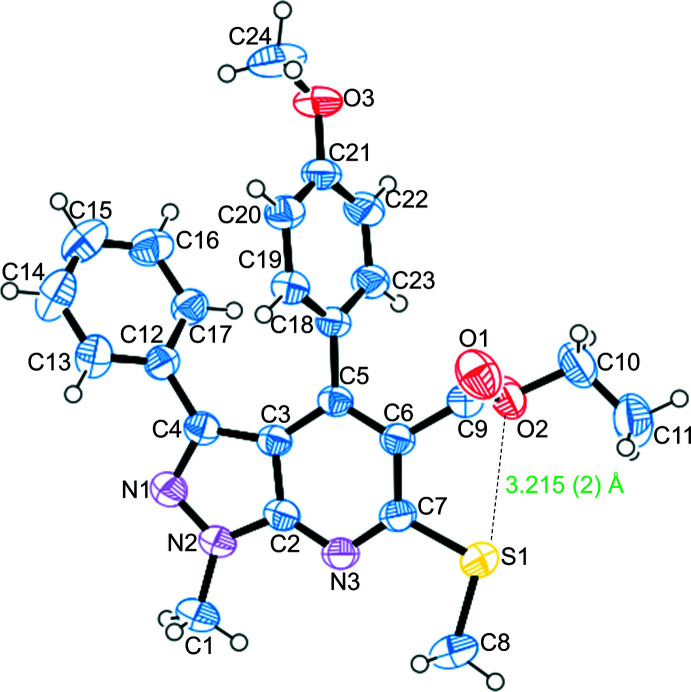
The mol­ecular structure of the title compound with the atom-numbering scheme and displacement ellipsoids drawn at the 50% probability level. The short intra­molecular S⋯O inter­action is indicated by a dashed line.

In the title compound (Fig. 1 ▸), the phenyl (–C_6_H_5_) group attached to the pyrazolo­pyridine moiety exhibits a (+)*anti*-periplanar conformation [C12—C4—N1—N2 = 178.47 (14)°] whereas the meth­oxy­phenyl (H_3_COC_6_H_4_–) group exhibits a (−)*anti*-clinal conformation [C3—C5—C18—C23 = −114.30 (19)°]. The thio­methyl (–SCH_3_) group fused to the pyrazolo­pyridine unit exhibits a (+)*anti*-periplanar conformation [C8—S1—C7—C6 = 175.47 (15)°]. The torsion angles involving the –SCH_3_ group differ from those for NUDWOB (Rao *et al.* 2020 ▸) because of the presence of the meth­oxy­phenyl (H_3_COC_6_H_4_–) group. The –COOC_2_H_5_ group attached to the pyrazolo­pyridine moiety has a (+)*anti*-periplanar conformation [N3—C7—C6—C9 = 176.44 (16)°]. Further, the methyl group attached to the pyrazole subunit is (+)*anti*-periplanar [C1—N2—N1—C4 = 178.78 (17)°] and it is attached to the pyridine ring showing a (+)*syn*-periplanar conformation [C1—N2—C2—N3 = 1.3 (3)°]. The fused pyrazole and pyridine rings in the title compound are not exactly planar, as in NUDWOB (Rao *et al.* 2020 ▸), subtending a dihedral angle of 1.76 (7)°. The dihedral angle between the planes of the benzene and pyrazolo­[3,4-*b*]pyridine rings is 44.8 (5)° and that between the meth­oxy­phenyl and pyrazolo­[3,4-*b*]pyridine rings is 63.86 (5)°.

## Supra­molecular features   

**Figure 2:**
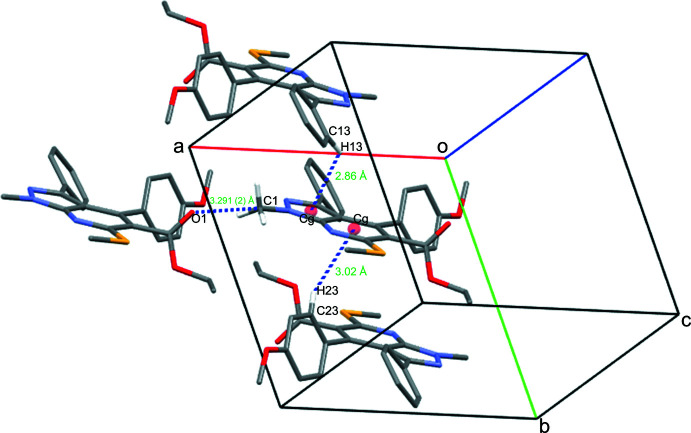
A view of the weak inter­molecular C13—H13⋯*Cg*(N1/N2/C2–C4), C23—H13⋯*Cg*(N3/C2/C3/C5–C7) and C1⋯O1 inter­actions in the title compound.

The cohesion of the crystal packing is influenced by two weak C—H⋯π (C13—H13⋯*Cg* and C23—H23⋯*Cg*) inter­actions (Table 1 ▸, Fig. 2 ▸) with distances of 2.86 and 3.02 Å, respectively. These distances agree with those described by Nishio (2011 ▸). A short inter­molecular C⋯O carbon=bonding stabilizing inter­action [C1⋯O1(*x* + 1, *y*, *z*) = 3.291 (2) Å] may also exist (Fig. 2 ▸) between the electrophilic carbon atom of the methyl group connected to the electronegative nitro­gen atom of the pyrazolo ring and the nucleophilic oxygen atom of the ester group of a neighbouring mol­ecule. However, the distance between C1 and O1 is marginally higher than the carbon-bonding distances (less than 3.22 Å, the sum of the van der Waals radii of carbon and oxygen atoms) proposed by Guru Row and co-workers (Thomas *et al.*, 2014 ▸). The distances between the methyl hydrogen atoms and the acceptor oxygen atom are C—H1*A*⋯O1 = 3.06, C—H1*B*⋯O1 = 3.04 and C—H1*C*⋯O1 = 3.22 Å, much longer than the hydrogen-bonding inter­actions (C—H⋯O = 2.90, 2.84 and 2.86 Å) noted by Thomas *et al.* (2014 ▸). Based on these observations, in addition to the C—H⋯π inter­actions, the short inter­molecular C⋯O carbon-bonding inter­action may also contribute to the cohesion of the title compound in the solid state.

## Database survey   

A similarity search of the Cambridge Structural Database (CSD, Version 5.40, update of March 2020; Groom *et al.*, 2016 ▸) was performed. The title compound, along with related structures obtained from the database search could be used for further structure-based virtual screening, ligand-based virtual screening, pharmacophore-based virtual screening and drug repurposing against various drug target proteins. The mol­ecules showing strong binding affinity towards drug target proteins might be considered potential lead candidates. In this study, the CSD search found five mol­ecules that are similar to title compound, namely FIZLEI (ethyl 2,7-di­amino-3,4-di­cyano-5-phenyl­pyrazolo­[1,5-*a*]pyridine-6-carboxyl­ate; Naik *et al.*, 2019 ▸), ALAFID [7-(2-meth­oxy­phen­yl)-2-phenyl­pyrazolo[1,5-*a*]pyridine; Wu *et al.*, 2016 ▸], DAWKAQ [2-(4-chloro­phen­yl)pyrazolo­[1,5-*a*]pyridin-3-yl(phen­yl)methanone; Ravi *et al.*, 2017 ▸], NADPIU [3-(4-chloro­phen­yl)pyrazolo­[1,5-*a*]pyridine; Wu *et al.*, 2016 ▸] and NUDWOB [ethyl 3-(4-chloro­phen­yl)-1,6-dimethyl-4-(methyl­sulfan­yl)-1*H*-pyrazolo­[3,4-*b*]pyridine-5-carboxyl­ate; Rao *et al.*, 2020 ▸]. The geometrical parameters of the –COOCH_2_CH_3_ substituent in the title compound are comparable with those reported for FIZLEI and NUDWOB. The bond distances for the thio­methyl and aryl moieties in the title compound are comparable with those of NUDWOB. Moreover, the bond lengths in the pyrazolo[3,4-*b*]pyridine unit of the title compound are comparable with those in NUDWOB, FIZLEI, ALAFID, DAWKAQ and NADPIU. The pyrazolo­[3,4-*b*]pyridine moiety (N1–N3/C2–C4/C5–C7) of the title compound is approximately planar, as is also observed for NUDWOB, FIZLEI, ALAFID, DAWKAQ and NADPIU. In the title compound, a short intra­molecular S⋯O contact of 3.215 (2) Å occurs, but this is not observed in FIZLEI, ALAFID, DAWKAQ, NADPIU and NUDWOB. Moreover, the inter­action distance [3.291 (2) Å] of the short inter­molecular C⋯O contact in the title compound is comparable with the C⋯O inter­action [3.424 (2) Å] in the structure of NUDWOB. Furthermore, as in the title compound, C—H⋯π inter­actions are observed in the crystal structures of ALAFID, DAWKAQ and NADPIU. The inter­action distance of these related structures ranges from 2.89 to 3.23 Å, comparable with the C—H⋯π inter­actions observed in the title compound.

## Synthesis and crystallization   

To a solution of 1-methyl-3-phenyl-1*H*-pyrazol-5-amine (100 mg, 0.57 mmol) and ethyl 2-(4-meth­oxy­benzo­yl)-3,3-bis­(methyl­thio)­acrylate (188 mg, 0.57 mmol) in toluene (3 mL), a catalytic amount of TFA (tri­fluoro­acetic acid) 30 mol % in toluene (3 mL) was added under an N_2_ atmosphere. The reaction mixture was refluxed for 24 h in an oil bath, the progress of the reaction being monitored by TLC using a mixture of hexane and ethyl acetate (9.9:0.1). After completion of the reaction, the mixture was loaded on a silica gel column (100–200 mesh, 15 cm × 1 cm) and eluted with increasing amounts of ethyl acetate in hexa­nes (1% to 5%) to obtain 186 mg (yield = 75%) of ethyl 6-(4-meth­oxy­phen­yl)-1-methyl-4-(methyl­thio)-3-phenyl-1*H*-pyrazolo­[3,4-*b*]pyridine-5-carboxyl­ate as a colourless crystalline solid; m.p. 406 K; *R*_f_ = 0.3 cm (hexa­ne: ethyl acetate 9.9:0.1). A sample suitable for single-crystal X-ray analysis was obtained by recrystallization from 2 mL of dry methanol.

## Refinement   

Crystal data, data collection and structure refinement details are summarized in Table 2 ▸. The hydrogen atoms were placed in calculated positions, with C—H = 0.93–0.97 Å and included in the final cycles of refinement using a riding model with *U*_iso_(H) = 1.2*U*_eq_(C) or 1.5*U*_eq_(C-meth­yl).

## Supplementary Material

Crystal structure: contains datablock(s) I. DOI: 10.1107/S2056989020008841/dx2029sup1.cif

Structure factors: contains datablock(s) I. DOI: 10.1107/S2056989020008841/dx2029Isup2.hkl

Supporting information file. DOI: 10.1107/S2056989020008841/dx2029Isup3.cml

CCDC reference: 2005976

Additional supporting information:  crystallographic information; 3D view; checkCIF report

## Figures and Tables

**Table 1:** Hydrogen-bond geometry (Å, °) *Cg*1 and *Cg*2 are the centroids of the N1/N2/C2–C4 and the N3/C2/C3/C5–C7 rings, respectively.

*D*—H⋯*A*	*D*—H	H⋯*A*	*D*⋯*A*	*D*—H⋯*A*
C13—H13⋯*Cg*1^i^	0.93	2.85	3.455 (2)	124
C23—H23⋯*Cg*2^ii^	0.93	3.02	3.738 (2)	136

Symmetry codes: (i) 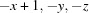; (ii) 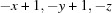.

**Table 2:** Experimental details

Crystal data	
Chemical formula	C_24_H_23_N_3_O_3_S
*M*_r_	433.51
Crystal system, space group	Triclinic, *P* 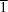
Temperature (K)	298
*a*, *b*, *c* (Å)	10.1911 (4), 10.7274 (6), 11.7692 (6)
α, β, γ (°)	98.550 (5), 105.632 (4), 111.015 (4)
*V* (Å^3^)	1112.75 (10)
*Z*	2
Radiation type	Mo *K*α
μ (mm^−1^)	0.18
Crystal size (mm)	0.75 × 0.44 × 0.42

Data collection	
Diffractometer	Agilent Xcalibur, Eos
Absorption correction	Multi-scan (*CrysAlis PRO*; Agilent, 2014 ▸)
*T*_min_, *T*_max_	0.959, 1.000
No. of measured, independent and observed [*I* > 2σ(*I*)] reflections	13297, 5161, 3672
*R*_int_	0.026
(sin θ/λ)_max_ (Å^−1^)	0.686

Refinement	
*R*[*F* ^2^ > 2σ(*F* ^2^)], *wR*(*F* ^2^), *S*	0.048, 0.168, 1.06
No. of reflections	5161
No. of parameters	284
H-atom treatment	H-atom parameters constrained
Δρ_max_, Δρ_min_ (e Å^−3^)	0.20, −0.26

Computer programs: *CrysAlis PRO* (Agilent, 2014 ▸), *SHELXT* (Sheldrick, 2015 ▸), *SHELXL2018* (Sheldrick, 2015 ▸), *ORTEP-3 for Windows* (Farrugia, 2012 ▸), *PLATON* (Spek, 2020 ▸) and *Mercury* (Macrae *et al.*, 2020 ▸).

## supplementary crystallographic information

### Crystal data

**Table:**

C_24_H_23_N_3_O_3_S	*Z* = 2
*M**_r_* = 433.51	*F*(000) = 456
Triclinic, *P*1	*D*_x_ = 1.294 Mg m^−^^3^
*a* = 10.1911 (4) Å	Mo *K*α radiation, λ = 0.71073 Å
*b* = 10.7274 (6) Å	Cell parameters from 3586 reflections
*c* = 11.7692 (6) Å	θ = 3.8–29.1°
α = 98.550 (5)°	µ = 0.18 mm^−^^1^
β = 105.632 (4)°	*T* = 298 K
γ = 111.015 (4)°	Block, colorless
*V* = 1112.75 (10) Å^3^	0.75 × 0.44 × 0.42 mm

### Data collection

**Table:**

Agilent Xcalibur, Eos diffractometer	5161 independent reflections
Radiation source: Enhance (Mo) X-ray Source	3672 reflections with *I* > 2σ(*I*)
Detector resolution: 15.9821 pixels mm^-1^	*R*_int_ = 0.026
ω scans	θ_max_ = 29.2°, θ_min_ = 3.8°
Absorption correction: multi-scan (CrysAlis PRO; Agilent, 2014)	*h* = −13→12
*T*_min_ = 0.959, *T*_max_ = 1.000	*k* = −13→14
13297 measured reflections	*l* = −15→15

### Refinement

**Table:**

Refinement on *F*^2^	Primary atom site location: structure-invariant direct methods
Least-squares matrix: full	Secondary atom site location: difference Fourier map
*R*[*F*^2^ > 2σ(*F*^2^)] = 0.048	Hydrogen site location: inferred from neighbouring sites
*wR*(*F*^2^) = 0.168	H-atom parameters constrained
*S* = 1.06	*w* = 1/[σ^2^(*F*_o_^2^) + (0.1*P*)^2^] where *P* = (*F*_o_^2^ + 2*F*_c_^2^)/3
5161 reflections	(Δ/σ)_max_ < 0.001
284 parameters	Δρ_max_ = 0.20 e Å^−^^3^
0 restraints	Δρ_min_ = −0.26 e Å^−^^3^

### Special details

**Table:**

Geometry. All esds (except the esd in the dihedral angle between two l.s. planes) are estimated using the full covariance matrix. The cell esds are taken into account individually in the estimation of esds in distances, angles and torsion angles; correlations between esds in cell parameters are only used when they are defined by crystal symmetry. An approximate (isotropic) treatment of cell esds is used for estimating esds involving l.s. planes.

### Fractional atomic coordinates and isotropic or equivalent isotropic displacement parameters (Å^2^)

**Table:**

	*x*	*y*	*z*	*U*_iso_*/*U*_eq_	
S1	0.67085 (6)	0.56012 (6)	0.41095 (5)	0.0647 (2)	
O2	0.38290 (15)	0.56954 (14)	0.22189 (15)	0.0618 (4)	
N3	0.72006 (14)	0.40967 (14)	0.23884 (14)	0.0456 (4)	
O3	−0.16778 (14)	0.10375 (17)	−0.27340 (14)	0.0716 (4)	
O1	0.26375 (16)	0.36781 (15)	0.25921 (15)	0.0716 (4)	
N2	0.74888 (15)	0.26571 (16)	0.08007 (15)	0.0507 (4)	
N1	0.66463 (15)	0.17775 (16)	−0.03455 (15)	0.0524 (4)	
C6	0.46748 (17)	0.39796 (17)	0.18528 (16)	0.0426 (4)	
C18	0.25937 (17)	0.25136 (17)	−0.01408 (15)	0.0403 (4)	
C5	0.41691 (17)	0.30623 (16)	0.07038 (15)	0.0397 (4)	
C23	0.2031 (2)	0.33918 (19)	−0.06467 (18)	0.0509 (4)	
H23	0.262169	0.434277	−0.040467	0.061*	
C19	0.16737 (17)	0.11066 (18)	−0.05037 (16)	0.0450 (4)	
H19	0.203616	0.050794	−0.017572	0.054*	
C4	0.52914 (18)	0.17844 (18)	−0.06206 (17)	0.0440 (4)	
C3	0.52331 (17)	0.26645 (16)	0.03830 (16)	0.0403 (4)	
C12	0.41458 (18)	0.09398 (18)	−0.18366 (16)	0.0443 (4)	
C21	−0.02998 (18)	0.1451 (2)	−0.18636 (17)	0.0507 (4)	
C20	0.02258 (18)	0.05683 (19)	−0.13439 (16)	0.0491 (4)	
H20	−0.038626	−0.037581	−0.155630	0.059*	
C7	0.61907 (18)	0.44751 (18)	0.26597 (17)	0.0451 (4)	
C17	0.3250 (2)	0.1467 (2)	−0.25396 (17)	0.0518 (4)	
H17	0.335347	0.236158	−0.223765	0.062*	
C22	0.0606 (2)	0.2860 (2)	−0.15032 (19)	0.0575 (5)	
H22	0.024819	0.345484	−0.184333	0.069*	
C1	0.9027 (2)	0.2863 (3)	0.1384 (2)	0.0737 (6)	
H1A	0.954270	0.370449	0.204079	0.110*	
H1B	0.952102	0.292919	0.079218	0.110*	
H1C	0.903503	0.209297	0.170462	0.110*	
C2	0.66831 (17)	0.32140 (18)	0.12734 (17)	0.0435 (4)	
C16	0.2213 (2)	0.0669 (2)	−0.36804 (19)	0.0632 (5)	
H16	0.161631	0.102596	−0.414423	0.076*	
C13	0.3980 (2)	−0.0386 (2)	−0.23167 (19)	0.0583 (5)	
H13	0.458611	−0.074552	−0.186802	0.070*	
C9	0.35959 (19)	0.44074 (19)	0.22766 (17)	0.0477 (4)	
C10	0.2937 (3)	0.6327 (2)	0.2651 (3)	0.0740 (7)	
H10A	0.202428	0.560492	0.264182	0.089*	
H10B	0.265806	0.685559	0.210502	0.089*	
C15	0.2054 (3)	−0.0656 (2)	−0.4137 (2)	0.0716 (6)	
H15	0.135537	−0.119091	−0.490858	0.086*	
C8	0.8584 (2)	0.5829 (3)	0.4825 (2)	0.0821 (7)	
H8A	0.859059	0.495191	0.489817	0.123*	
H8B	0.900377	0.646868	0.562541	0.123*	
H8C	0.917043	0.619009	0.433710	0.123*	
C11	0.3801 (3)	0.7253 (3)	0.3911 (3)	0.0868 (8)	
H11A	0.402829	0.671847	0.445827	0.130*	
H11B	0.321725	0.769022	0.416900	0.130*	
H11C	0.471812	0.795051	0.392304	0.130*	
C24	−0.2684 (2)	−0.0390 (3)	−0.3089 (3)	0.0881 (8)	
H24A	−0.228284	−0.093559	−0.348676	0.132*	
H24B	−0.363754	−0.052474	−0.364364	0.132*	
H24C	−0.280979	−0.067455	−0.237465	0.132*	
C14	0.2926 (3)	−0.1182 (2)	−0.3453 (2)	0.0756 (7)	
H14	0.280866	−0.208232	−0.375564	0.091*	

### Atomic displacement parameters (Å^2^)

**Table:**

	*U*^11^	*U*^22^	*U*^33^	*U*^12^	*U*^13^	*U*^23^
S1	0.0602 (3)	0.0687 (4)	0.0507 (3)	0.0333 (3)	−0.0005 (2)	−0.0037 (3)
O2	0.0566 (7)	0.0531 (8)	0.0840 (11)	0.0294 (6)	0.0309 (7)	0.0137 (7)
N3	0.0379 (7)	0.0429 (8)	0.0482 (9)	0.0157 (6)	0.0063 (6)	0.0105 (7)
O3	0.0436 (7)	0.0890 (11)	0.0611 (10)	0.0256 (7)	−0.0054 (7)	0.0109 (8)
O1	0.0659 (9)	0.0703 (10)	0.0955 (12)	0.0307 (7)	0.0459 (9)	0.0313 (9)
N2	0.0348 (7)	0.0594 (9)	0.0538 (10)	0.0211 (6)	0.0110 (7)	0.0093 (8)
N1	0.0436 (8)	0.0610 (10)	0.0527 (10)	0.0239 (7)	0.0166 (7)	0.0112 (8)
C6	0.0380 (8)	0.0409 (9)	0.0459 (10)	0.0173 (7)	0.0096 (7)	0.0109 (8)
C18	0.0347 (8)	0.0456 (9)	0.0397 (9)	0.0177 (7)	0.0101 (7)	0.0123 (7)
C5	0.0366 (8)	0.0395 (8)	0.0419 (9)	0.0163 (6)	0.0099 (7)	0.0144 (7)
C23	0.0478 (9)	0.0459 (10)	0.0541 (11)	0.0218 (8)	0.0078 (8)	0.0129 (8)
C19	0.0405 (9)	0.0471 (9)	0.0474 (10)	0.0181 (7)	0.0137 (8)	0.0167 (8)
C4	0.0400 (8)	0.0466 (9)	0.0459 (10)	0.0178 (7)	0.0155 (7)	0.0141 (8)
C3	0.0359 (8)	0.0391 (8)	0.0440 (10)	0.0155 (6)	0.0101 (7)	0.0137 (7)
C12	0.0414 (8)	0.0489 (10)	0.0404 (10)	0.0150 (7)	0.0174 (7)	0.0098 (8)
C21	0.0370 (9)	0.0682 (12)	0.0419 (10)	0.0220 (8)	0.0081 (8)	0.0120 (9)
C20	0.0388 (9)	0.0520 (10)	0.0486 (11)	0.0124 (7)	0.0131 (8)	0.0130 (8)
C7	0.0411 (8)	0.0406 (9)	0.0452 (10)	0.0154 (7)	0.0059 (7)	0.0097 (7)
C17	0.0512 (10)	0.0547 (11)	0.0459 (11)	0.0210 (8)	0.0145 (8)	0.0111 (9)
C22	0.0532 (10)	0.0623 (12)	0.0574 (12)	0.0329 (9)	0.0073 (9)	0.0182 (10)
C1	0.0404 (10)	0.0916 (16)	0.0792 (16)	0.0328 (10)	0.0088 (10)	0.0052 (13)
C2	0.0370 (8)	0.0442 (9)	0.0484 (10)	0.0180 (7)	0.0112 (7)	0.0149 (8)
C16	0.0566 (11)	0.0755 (14)	0.0472 (12)	0.0219 (10)	0.0108 (9)	0.0167 (11)
C13	0.0737 (12)	0.0529 (11)	0.0523 (12)	0.0292 (9)	0.0241 (10)	0.0152 (9)
C9	0.0413 (9)	0.0508 (10)	0.0460 (10)	0.0199 (7)	0.0098 (8)	0.0077 (8)
C10	0.0638 (13)	0.0697 (14)	0.1010 (19)	0.0400 (11)	0.0353 (13)	0.0147 (13)
C15	0.0773 (14)	0.0637 (14)	0.0447 (12)	0.0073 (11)	0.0135 (11)	0.0049 (10)
C8	0.0566 (12)	0.0955 (18)	0.0603 (15)	0.0251 (12)	−0.0075 (11)	−0.0043 (13)
C11	0.1061 (19)	0.0984 (19)	0.0812 (19)	0.0613 (16)	0.0479 (16)	0.0204 (16)
C24	0.0442 (11)	0.1039 (19)	0.0718 (17)	0.0056 (11)	−0.0057 (11)	0.0133 (15)
C14	0.1098 (18)	0.0521 (12)	0.0520 (13)	0.0261 (12)	0.0237 (13)	0.0066 (10)

### Geometric parameters (Å, º)

**Table:**

S1—C7	1.7590 (19)	C12—C17	1.392 (3)
S1—C8	1.777 (2)	C21—C22	1.383 (3)
O2—C9	1.331 (2)	C21—C20	1.385 (3)
O2—C10	1.460 (2)	C20—H20	0.9300
N3—C7	1.328 (2)	C17—C16	1.378 (3)
N3—C2	1.340 (2)	C17—H17	0.9300
O3—C21	1.364 (2)	C22—H22	0.9300
O3—C24	1.423 (3)	C1—H1A	0.9600
O1—O1	0.000 (4)	C1—H1B	0.9600
O1—C9	1.199 (2)	C1—H1C	0.9600
N2—C2	1.356 (2)	C16—C15	1.377 (3)
N2—N1	1.365 (2)	C16—H16	0.9300
N2—C1	1.450 (2)	C13—C14	1.378 (3)
N1—C4	1.334 (2)	C13—H13	0.9300
C6—C5	1.388 (2)	C10—C11	1.481 (3)
C6—C7	1.429 (2)	C10—H10A	0.9700
C6—C9	1.503 (2)	C10—H10B	0.9700
C18—C19	1.385 (2)	C15—C14	1.366 (4)
C18—C23	1.393 (2)	C15—H15	0.9300
C18—C5	1.483 (2)	C8—H8A	0.9600
C5—C3	1.416 (2)	C8—H8B	0.9600
C23—C22	1.377 (3)	C8—H8C	0.9600
C23—H23	0.9300	C11—H11A	0.9600
C19—C20	1.386 (2)	C11—H11B	0.9600
C19—H19	0.9300	C11—H11C	0.9600
C4—C3	1.427 (2)	C24—H24A	0.9600
C4—C12	1.479 (2)	C24—H24B	0.9600
C3—C2	1.409 (2)	C24—H24C	0.9600
C12—C13	1.381 (3)	C14—H14	0.9300
			
C7—S1—C8	102.08 (11)	H1A—C1—H1B	109.5
C9—O2—C10	118.64 (16)	N2—C1—H1C	109.5
C7—N3—C2	114.13 (14)	H1A—C1—H1C	109.5
C21—O3—C24	117.99 (17)	H1B—C1—H1C	109.5
C2—N2—N1	111.38 (13)	N3—C2—N2	125.09 (15)
C2—N2—C1	127.81 (17)	N3—C2—C3	128.10 (15)
N1—N2—C1	120.77 (16)	N2—C2—C3	106.80 (15)
C4—N1—N2	106.91 (14)	C15—C16—C17	120.3 (2)
C5—C6—C7	120.93 (15)	C15—C16—H16	119.9
C5—C6—C9	119.54 (14)	C17—C16—H16	119.9
C7—C6—C9	119.47 (16)	C14—C13—C12	120.77 (19)
C19—C18—C23	118.28 (15)	C14—C13—H13	119.6
C19—C18—C5	120.91 (14)	C12—C13—H13	119.6
C23—C18—C5	120.70 (15)	O1—C9—O2	124.91 (16)
C6—C5—C3	116.49 (14)	O1—C9—O2	124.91 (16)
C6—C5—C18	121.92 (14)	O1—C9—C6	124.73 (17)
C3—C5—C18	121.59 (15)	O1—C9—C6	124.73 (17)
C22—C23—C18	120.38 (17)	O2—C9—C6	110.33 (15)
C22—C23—H23	119.8	O2—C10—C11	110.43 (19)
C18—C23—H23	119.8	O2—C10—H10A	109.6
C18—C19—C20	121.62 (15)	C11—C10—H10A	109.6
C18—C19—H19	119.2	O2—C10—H10B	109.6
C20—C19—H19	119.2	C11—C10—H10B	109.6
N1—C4—C3	110.06 (15)	H10A—C10—H10B	108.1
N1—C4—C12	118.69 (16)	C14—C15—C16	119.8 (2)
C3—C4—C12	131.25 (14)	C14—C15—H15	120.1
C2—C3—C5	116.67 (15)	C16—C15—H15	120.1
C2—C3—C4	104.82 (14)	S1—C8—H8A	109.5
C5—C3—C4	138.47 (15)	S1—C8—H8B	109.5
C13—C12—C17	118.49 (17)	H8A—C8—H8B	109.5
C13—C12—C4	120.04 (16)	S1—C8—H8C	109.5
C17—C12—C4	121.44 (16)	H8A—C8—H8C	109.5
O3—C21—C22	115.77 (17)	H8B—C8—H8C	109.5
O3—C21—C20	124.66 (17)	C10—C11—H11A	109.5
C22—C21—C20	119.56 (16)	C10—C11—H11B	109.5
C21—C20—C19	119.30 (17)	H11A—C11—H11B	109.5
C21—C20—H20	120.4	C10—C11—H11C	109.5
C19—C20—H20	120.4	H11A—C11—H11C	109.5
N3—C7—C6	123.66 (17)	H11B—C11—H11C	109.5
N3—C7—S1	118.89 (13)	O3—C24—H24A	109.5
C6—C7—S1	117.42 (13)	O3—C24—H24B	109.5
C16—C17—C12	120.30 (19)	H24A—C24—H24B	109.5
C16—C17—H17	119.9	O3—C24—H24C	109.5
C12—C17—H17	119.9	H24A—C24—H24C	109.5
C23—C22—C21	120.80 (17)	H24B—C24—H24C	109.5
C23—C22—H22	119.6	C15—C14—C13	120.3 (2)
C21—C22—H22	119.6	C15—C14—H14	119.8
N2—C1—H1A	109.5	C13—C14—H14	119.8
N2—C1—H1B	109.5		
			
C2—N2—N1—C4	0.92 (19)	C5—C6—C7—S1	−178.50 (13)
C1—N2—N1—C4	178.78 (17)	C9—C6—C7—S1	−1.3 (2)
C7—C6—C5—C3	−0.5 (2)	C8—S1—C7—N3	−2.36 (18)
C9—C6—C5—C3	−177.72 (15)	C8—S1—C7—C6	175.47 (15)
C7—C6—C5—C18	178.87 (14)	C13—C12—C17—C16	0.8 (3)
C9—C6—C5—C18	1.6 (2)	C4—C12—C17—C16	178.66 (16)
C19—C18—C5—C6	−117.54 (18)	C18—C23—C22—C21	0.9 (3)
C23—C18—C5—C6	66.4 (2)	O3—C21—C22—C23	−178.79 (18)
C19—C18—C5—C3	61.8 (2)	C20—C21—C22—C23	0.9 (3)
C23—C18—C5—C3	−114.30 (19)	C7—N3—C2—N2	−178.44 (16)
C19—C18—C23—C22	−1.3 (3)	C7—N3—C2—C3	0.3 (2)
C5—C18—C23—C22	174.93 (17)	N1—N2—C2—N3	178.93 (15)
C23—C18—C19—C20	−0.2 (3)	C1—N2—C2—N3	1.3 (3)
C5—C18—C19—C20	−176.43 (16)	N1—N2—C2—C3	0.00 (19)
N2—N1—C4—C3	−1.47 (18)	C1—N2—C2—C3	−177.67 (18)
N2—N1—C4—C12	178.47 (14)	C5—C3—C2—N3	−1.5 (3)
C6—C5—C3—C2	1.5 (2)	C4—C3—C2—N3	−179.75 (16)
C18—C5—C3—C2	−177.89 (14)	C5—C3—C2—N2	177.41 (14)
C6—C5—C3—C4	178.94 (18)	C4—C3—C2—N2	−0.86 (17)
C18—C5—C3—C4	−0.4 (3)	C12—C17—C16—C15	−0.2 (3)
N1—C4—C3—C2	1.46 (18)	C17—C12—C13—C14	−1.6 (3)
C12—C4—C3—C2	−178.47 (17)	C4—C12—C13—C14	−179.45 (18)
N1—C4—C3—C5	−176.19 (19)	O1—O1—C9—O2	0.00 (13)
C12—C4—C3—C5	3.9 (3)	O1—O1—C9—C6	0.00 (9)
N1—C4—C12—C13	42.4 (2)	C10—O2—C9—O1	4.7 (3)
C3—C4—C12—C13	−137.69 (19)	C10—O2—C9—O1	4.7 (3)
N1—C4—C12—C17	−135.43 (18)	C10—O2—C9—C6	−177.05 (17)
C3—C4—C12—C17	44.5 (3)	C5—C6—C9—O1	73.3 (2)
C24—O3—C21—C22	−176.7 (2)	C7—C6—C9—O1	−104.0 (2)
C24—O3—C21—C20	3.6 (3)	C5—C6—C9—O1	73.3 (2)
O3—C21—C20—C19	177.29 (17)	C7—C6—C9—O1	−104.0 (2)
C22—C21—C20—C19	−2.4 (3)	C5—C6—C9—O2	−104.97 (18)
C18—C19—C20—C21	2.1 (3)	C7—C6—C9—O2	77.8 (2)
C2—N3—C7—C6	0.9 (2)	C9—O2—C10—C11	99.1 (2)
C2—N3—C7—S1	178.58 (12)	C17—C16—C15—C14	0.3 (3)
C5—C6—C7—N3	−0.8 (3)	C16—C15—C14—C13	−1.0 (4)
C9—C6—C7—N3	176.44 (16)	C12—C13—C14—C15	1.7 (3)

### Hydrogen-bond geometry (Å, º)

*Cg*1 and *Cg*2 are the centroids of the N1/N2/C2–C4 and the N3/C2/C3/C5–C7 rings, respectively.

**Table:**

*D*—H···*A*	*D*—H	H···*A*	*D*···*A*	*D*—H···*A*
C13—H13···*Cg*1^i^	0.93	2.85	3.455 (2)	124
C23—H23···*Cg*2^ii^	0.93	3.02	3.738 (2)	136

Symmetry codes: (i) −*x*+1, −*y*, −*z*; (ii) −*x*+1, −*y*+1, −*z*.
